# The *Neisseria gonorrhoeae* Obg protein is an essential ribosome-associated GTPase and a potential drug target

**DOI:** 10.1186/s12866-015-0453-1

**Published:** 2015-06-30

**Authors:** Ryszard A. Zielke, Igor H. Wierzbicki, Benjamin I. Baarda, Aleksandra E. Sikora

**Affiliations:** Department of Pharmaceutical Sciences, College of Pharmacy, Oregon State University, 433 Weniger Hall, 103 SW Memorial Pl, Corvallis, OR 97330 USA

**Keywords:** *Neisseria gonorrhoeae*, Drug resistance, Obg proteins, GTPase, Drug target, Mant guanine nucleotides

## Abstract

**Background:**

*Neisseria gonorrhoeae* (GC) is a Gram-negative pathogen that most commonly infects mucosal surfaces, causing sexually transmitted urethritis in men and endocervicitis in women. Serious complications associated with these infections are frequent and include pelvic inflammatory disease, ectopic pregnancy, and infertility. The incidence of gonorrhea cases remains high globally while antibiotic treatment options, the sole counter measures against gonorrhea, are declining due to the remarkable ability of GC to acquire resistance. Evaluating of potential drug targets is essential to provide opportunities for developing antimicrobials with new mechanisms of action. We propose the GC Obg protein, belonging to the Obg/CgtA GTPase subfamily, as a potential target for the development of therapeutic interventions against gonorrhea, and in this study perform its initial functional and biochemical characterization.

**Results:**

We report that NGO1990 encodes Obg protein, which is an essential factor for GC viability, associates predominantly with the large 50S ribosomal subunit, and is stably expressed under conditions relevant to infection of the human host. The anti-Obg antisera cross-reacts with a panel of contemporary GC clinical isolates, demonstrating the ubiquitous nature of Obg. The cellular levels of Obg reach a maximum in the early logarithmic phase and remain constant throughout bacterial growth. The in vitro binding and hydrolysis of the fluorescent guanine nucleotide analogs mant-GTP and mant-GDP by recombinant wild type and T192AT193A mutated variants of Obg are also assessed.

**Conclusions:**

Characterization of the GC Obg at the molecular and functional levels presented herein may facilitate the future targeting of this protein with small molecule inhibitors and the evaluation of identified lead compounds for bactericidal activity against GC and other drug-resistant bacteria.

**Electronic supplementary material:**

The online version of this article (doi:10.1186/s12866-015-0453-1) contains supplementary material, which is available to authorized users.

## Background

*Neisseria gonorrhoeae* (GC) is a Gram-negative bacterium and a human-specific pathogen that causes gonorrhea. This sexually transmitted disease remains a global health burden. The World Health Organization estimated 106.1 million new cases in adults in 2008, which was a 21 % increase compared to 2005 [1]. The disease usually manifests as cervicitis, urethritis, proctitis, conjunctivis, or pharyngitis. A significant proportion of women (≥50 %) and some men (≤10 %) undergo asymptomatic infections and therefore many cases remain undiagnosed [2]. Untreated or inadequately treated gonorrhea often has serious long-term health consequences including endometritis, pelvic inflammatory disease, ectopic pregnancy, epididymitis, and infertility [2–4]. The serious sequelae of gonorrhea are exacerbated by a significant increase of the risk of HIV acquisition [5]. Pharmaceutical interventions against GC infections are limited to antibiotic regimens, as a preventive anti-gonorrhea vaccine does not exist. Antibiotic therapies, however, have been continually challenged by the remarkable ability of the bacteria to acquire and retain resistance [6]. Treatment failures associated with the current emergence of GC with decreased susceptibility to the last effective treatment option, third-generation cephalosporins, are concerning and emphasize the pressing need for the development of alternative antimicrobial strategies to combat drug-resistant gonorrhea [1, 6–14].

Here, we focus on biochemical and functional characterization of the GC homolog of conserved bacterial Obg GTPases, NGO1990 (henceforth Obg_GC_), as a target for the discovery of anti-gonorrhea compounds. Obg proteins (also recognized as YhbZ or CgtA) belong to the OBG-HflX superfamily within the TRAFAC (translational factors) class of P-loop (phosphate-binding loop) GTPases (reviewed in [15, 16]). The OBG family is comprised of four subfamilies: Obg, Nog1, DRG, and YchF. The family exists in all three domains of life with bacteria possessing Obg and YchF, archea having two Obg proteins and YchF, and eukaryotes commonly encoding four Obg proteins and YchF [15–18]. Structurally, the bacterial Obg proteins contain highly conserved N-terminal- and central-domains, and C-terminal domain that can vary in length and sequence, or as in *Chlamydia*, may even be absent [16, 19]. The N-terminal domain is glycine-rich and has a unique fold, the Obg fold [20]. The signature GTP-binding domain shares overall topology with the small Ras-like GTPases, but the biochemical features of the Obg proteins are distinct from those of eukaryotic Ras-like proteins [21–26].

The name Obg originates from *spo0B*-associated GTP-binding protein of *Bacillus subtilis*, in which the *obg* gene was identified as a part of the *spo0B* operon [27]. Since its identification in 1989, Obg homologs have been demonstrated to be essential for viability not only in *B. subtilis*, but also *Streptomyces coelicolor*, *Staphylococcus pneumoniae*, *S. aureus*, *Haemophilus influenzae*, *Caulobacter crescentus*, *Escherichia coli*, *Vibrio harveyi* and *V. cholerae* [27–33]. These findings strongly suggest that Obg proteins are crucial for the survival of both gram-positive and gram-negative bacteria. The depletion of cellular Obg levels results in species-specific pleiotropic effects on bacterial physiology, including alterations of ribosome maturation and DNA synthesis; cell division and morphology; and induction of general, as well as (p)ppGpp-mediated stringent stress responses [15, 16, 34]. Growing lines of evidence support the link between Obg and ribosome function. Obg predominantly associates with the 50S ribosomal particles, and long-term Obg depletion results in reduced levels of 70S monosomes, ribosomal proteins S1, S14, S21 and L10, as well as a perturbed polyribosome profile [17, 22, 32, 35–37]. Recent studies suggest that in *E. coli* cultured under standard laboratory growth conditions, Obg acts as a checkpoint in the final steps of 50S subunit assembly and, via an interplay with (p)ppGpp, might modulate the production of large ribosomal particle in response to environmental cues [34]. Introduction of the temperature-sensitive variant of *obg*, G80E, in *C. crescentus* caused a decline in both the growth rate and the amount of 50S subunits, even under permissive conditions [38]. Additional ribosome defects were not observed in non-permissive temperatures; however, the bacteria rapidly halted cell cycle progression and lost viability. Thus, the essential nature of Obg likely does not result directly from its function in the late stages of 50S subunit assembly [15, 38]. Obg might provide a key molecular nexus between different metabolic pathways to regulate cellular processes in response to the energy status of the cell [16]. All Obg proteins characterized to date bind GDP and GTP, and display relatively slow GTP hydrolysis, which can be moderately stimulated in the presence of purified 50S ribosomal particles [22, 24–26, 34, 39]. The mechanistic insights into how Obg participates in different metabolic paths and stress responses remain to be elucidated. Nevertheless, the Obg proteins appear to be promising molecular targets for the development of broad-spectrum antibiotics against drug-resistant bacterial infections because of their essential nature, conservation, and strong link with pivotal physiological processes.

## Results and discussion

### Local gene context and Obg_GC_ domain architecture

In virtually all bacteria the *obg* gene has been reported to be physically linked to *rplU* and *rpmA*, encoding 50S ribosomal proteins L21 and L27, respectively [15]. The inspection of the genetic organization of the *obg* region in available completed genome sequences of *Neisseriaceae*, however, revealed that this arrangement is not followed. In GC strain FA1090, the putative protein NGO1990, annotated as Obg, is the last ORF in a cluster comprised of NGO1989 and NGO1988. NGO1989 is a hypothetical protein present also in GC DGI18 and PID24-1, whereas NGO1988, encoding a homolog of the rRNA small subunit methyltransferase I, is located upstream of *obg* in other *Neisseriaceae*.

**Fig. 1:**
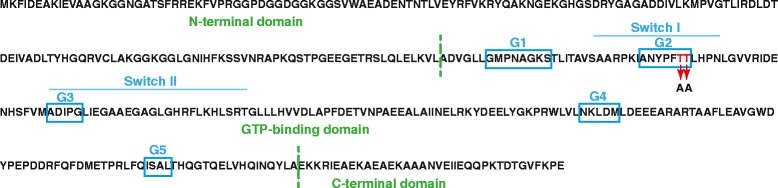
Obg_GC_ domain architecture. The individual structural domains of Obg_GC_ are shown in green. The N-terminal domain (amino acids 3–158) is glycine-rich. The central, GTP-binding domain (residues 160–348) includes two switch elements (switch I and switch II) and five conserved G motifs (G1-G5; indicated in blue boxes). The C-terminal domain contains clusters of acidic residues. The conserved T192 and T193 residues within the G2 motifs and introduced substitutions are designated in red

Analysis of the predicted amino acid sequence of NGO1990 from FA1090 revealed a typical structure of Obg GTPases (Fig. 1) and significant similarities to other Obg proteins (Table 1). The N-terminal domain of Obg_GC_ (amino acids 3–158) contains 26 glycine residues, similar to the *B. subtilis* Obg [20]. The central, GTP-binding domain (residues 160–348), includes five conserved G motifs (G1-G5) and two switch elements (switch I and switch II) that determine the active or inactive state of the G protein [15, 16]. As expected, the C-terminal domain shows the lowest conservation when compared to Obg homologs from different bacterial species (Additional file 1: Figure S1). Nevertheless, this region of Obg_GC_ contains clusters of acidic residues, a feature characteristic of Obg proteins [17, 33]. This charged C-terminus has been shown to be important for Obg association with 50S ribosomal particles in *C. crescentus* as well as GTP and GDP binding in *V. harveyi* [17, 26].

**Table 1:** Comparison of the amino acid sequences of the *N. gonorrhoeae* Obg protein with Obg homologs

Organism	Accession number	Protein length	Region aligned	% identity	% similarity
*Neisseria gonorrhoeae NCCP11945*	B4RQP4	384	1-384	100	100
*Neisseria meningitidis MC58*	Q9JXE5	384	1-384	98	98
*Neisseria lactamica* 020-06	E4ZAV0	384	1-384	97	97
*Neisseria weaveri LMG 5135*	G2DJV3	384	1-384	85	91
*Escherichia coli K12*	P42641	390	1-344	56	71
*Caulobacter crescentus NA1000/CB15N*	B8GYI7	354	1-320	52	67
*Bacillus subtilis 168*	P20964	428	2-328	49	68
*Chlamydia trachomatis D/UW-3/Cx*	O84423	335	2-335	43	62
*Homo sapiens*	Q9H4K7*	406	72-364	40	58
*Homo sapiens*	A4D1E9**	308	77-293	35	52
*Saccharomyces cerevisiae 204508*	P38860	518	295-488	40	61

*ObgH1 (GTP binding protein 5, GTBP5; mitochondrial ribosome-associated GTPase 2, MTG2)

**ObgH2 (GTP binding protein 10, GTPBP10)

### Purification of Obg variants and evaluation of anti-Obg_GC_ antisera

**Fig. 2:**
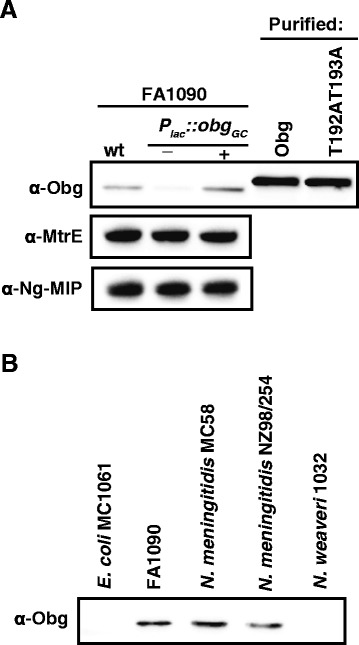
Validation of polyclonal rabbit anti-Obg_GC_ antisera. (**a**) The polyclonal rabbit anti-Obg_GC_ antibodies were used to probe the whole-cell lysates derived from wild type FA1090 and isogenic P_*lac*_::*obg*_*GC*_ as well as purified variants of Obg_GC_. The bacteria were harvested following 2 h of growth in GCBL with (+) and without (−) 100 μM IPTG, and the samples were matched by equivalent OD_600_ units. Purified recombinant proteins (40 ng) include wild type Obg_GC_ with N-terminal 6 × His tag, (N-His-Obg_GC_) and N-His-Obg_GC_ with T192AT193A substitutions. (**b**) Samples of whole-cell lysates derived from various *Neisseria* species, as indicated, were harvested from GCB and matched by equivalent OD_600_ units. All samples were separated in 4-20 % Mini-PROTEAN TGX precast gels, the proteins were transferred onto the nitrocellulose membrane and probed with polyclonal rabbit anti-Obg_GC_ antisera raised against N-His-Obg_GC_

To begin the characterization of Obg_GC_, N- and C-terminally His-tagged versions of the wild type NGO1990, N-His-Obg_GC_ and C-His-Obg_GC_ (respectively), were overexpressed in *E. coli* BL21(DE3), and purified. The purified recombinant N-His-Obg_GC_ was subsequently used to obtain polyclonal rabbit anti-Obg_GC_ antisera. The antisera specifically recognized both the native and recombinant versions of Obg_GC_ (Fig. 2a). The purified proteins migrated in SDS-PAGE more slowly than the native protein and accordingly with the deduced molecular mass of Obg_GC_ (41.998 kDa) with the addition of the histidine epitope. Further, the antibodies cross-reacted with Obg homologs in the *N. meningitidis* serogroup B strains MC58 and NZ98/254 but failed to recognize Obg from *N. weaveri* and *E. coli* despite their 85 and 56 % identity to Obg_GC_, respectively (Fig. 2b and Table 1). It is possible that the anti-Obg_GC_ antisera bind to the highly variable C-terminal domain of Obg_GC_.

### Obg_GC_ binds GTP and GDP

**Fig. 3:**
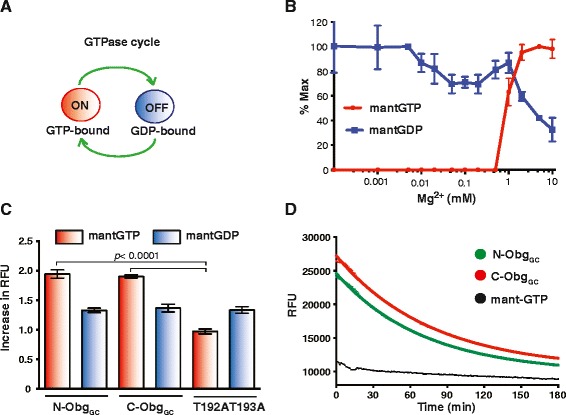
Biochemical properties of Obg_GC_. **a** GTPase cycle. Obg GTPases oscillate between active (ON, GTP-bound) and inactive (OFF, GDP-bound) states. **b** Obg_GC_ binds mant-GTP and mant-GDP with guanine nucleotide-specific Mg^2+^ dependence. Binding of mant-GTP (*red circles*) and mant-GDP (*blue squares*) to N-His-Obg_GC_ was assessed in the presence of varying concentrations of Mg^2+^. The averages with SEM from three independent experiments are shown. **c** Increase in Relative Fluorescence Units (RFU) of mant-GTP (*red bars*) and mant-GDP (*blue bars*) upon addition of different Obg_GC_ variants: recombinant wild type Obg_GC_ with N- and C-terminal 6 × His tag (N-His-Obg_GC_ and C-His-Obg_GC_, respectively), and N-His-Obg_GC_ with T192AT193A substitutions. The data shows averages with corresponding SEM of at least eight experiments performed on separate occasions. **d** Hydrolysis of mant-GTP by N-His-Obg_GC_ (*green*) and C-His-Obg_GC_ (*red*) was monitored by recording the decrease in fluorescence that is coupled to the conversion of mant-GTP-Obg to mant-GDP-Obg complexes. Data from at least four experiments were fitted to a single exponential decay equation. The fluorescence intensity of the mant-GTP in the absence of protein served as a control and is shown in black

GTPases cycle between being turned “on” in the GTP-bound state and turned “off” in the GDP-bound state (Fig. 3a). In each state, G proteins undergo conformational changes and downstream effectors sense the GTP-bound protein complexes. Switch-off involves the exchange of GTP for GDP or hydrolysis of the γ-phosphate of GTP. The fluorescent N-methyl-3’-*O*-anthranoyl (mant) guanine nucleotide analogs, mant-GTP and mant-GDP, have been widely utilized for examining the nucleotide binding and GTP hydrolysis of various G-proteins including Obg homologs. The highly environmentally sensitive fluorescence of the mant group enables detection of nucleotide-protein interaction [22, 24, 26, 38, 40–43]. The binding of GTP to Obg requires the presence of physiological Mg^2+^ concentrations in *C. crescentus*, *E. coli,* and *V. harveyi*, whereas Obg-GDP complexes form over a wide range of Mg^2+^ concentrations [22, 24, 26]. The total intracellular Mg^2+^ content is about 100 mM in *E. coli* and *B. subtilis* and includes bound and free Mg^2+^, with the latter ranging from 1–5 mM [44–46].

To determine whether Obg_GC_ requires Mg^2+^ to optimally bind mant-nucleotides, N-His-Obg_GC_ was incubated with increasing concentrations of Mg^2+^ and either mant-GTP or mant-GDP. The Obg_GC_ binding profiles obtained for both nucleotides differed noticeably similarly to that observed for other Obg family members [22, 24, 26]. The optimal formation of mant-GTP-Obg_GC_ complexes occurred between 5 and 10 mM Mg^2+^, as indicated by maximal fluorescence (Fig. 3b, red circles). The binding of mant-GDP to Obg_GC_ did not require Mg^2+^ and was inhibited at above 1 mM concentrations (Fig. 3b, blue squares).

Subsequently, binding of mant-GTP and mant-GDP was assessed for both N-and C-His-Obg_GC_, as addition of a six-histidine epitope to the C-terminus of the *V. harveyi* Obg completely abolished interaction with GTP and resulted in a weak binding of GDP [26]. Likewise, the *C. crescentus* Obg containing influenza virus hemagglutynin tag demonstrated a reduction in protein function [17]. In contrast, the C-His-Obg_GC_ showed very similar properties to N-His-Obg_GC_ (Fig. 3c). Binding of either variants of Obg_GC_ to mant-GTP and mant-GDP led to 1.9- and 1.3-fold enhancement in mant-nucleotide fluorescence, respectively. These results suggest that subtle perturbations to the C-terminus of Obg_GC_ are not detrimental to protein function. The *C. abortus* Obg naturally lacks the C-terminal domain, yet the protein is a functional GTPase, with similar activity to other Obg proteins, and binds to the 50S large ribosomal particle [19].

### mantGTP hydrolysis by Obg_GC_

We next examined the GTPase activity of purified N-His-Obg_GC_ and C-His-Obg_GC_ by monitoring the decrease in fluorescence that is associated with the single-turnover conversion of bound mant-GTP to bound mant-GDP (Fig. 3d). The peak of fluorescence was recorded for 3 h at 1 min intervals. The reduction in fluorescence was fitted to a single exponential decay with a first-order rate constant, *k*_*h*_, of 2.3 × 10^−4^ s^−1^and 2.1 × 10^−4^ s^−1^, or half life (T_1/2_) of 49.1 min and 53.2 min, for N-His-Obg_GC_ and C-His-Obg_GC_, respectively. Therefore, the GTP hydrolysis rates of both Obg_GC_ variants are very similar and are approximately twenty times slower than that of the *V. harveyi* Obg and two-fold slower than *C. crescentus* and *E. coli* Obg proteins, respectively [22, 24, 26]. These differences may reflect distinct Obg control or function in distantly related bacterial species.

### Alteration of switch I element of Obg_GC_ abolishes GTP but not GDP binding abilities

The two adjacent threonine residues, T192 and T193, which coordinate Mg^2+^, are ubiquitously present within the G2 domain of Obg proteins (Fig. 1). However, their function has been assessed only in *C. crescentus* [47]. To address their importance for guanine nucleotide binding in Obg_GC_, the double T192AT193A mutant protein with N-terminal-His epitope was constructed and purified. Compared with the wild type Obg_GC_, the mutated protein exhibited completely impaired mant-GTP binding, whereas a 1.3-fold increase in fluorescence was observed in the presence of mant-GDP, indicating unaffected formation of Obg-GDP complexes (Fig. 3c). A similar effect was observed in the *C. crescentus* Obg, and T193 was identified as the pivotal residue. The *obg* T193A allele was not able to support *C. crescentus* growth*,* which demonstrated that the Obg GTPase activity was a prerequisite for cell viability [47].

### Depletion of Obg_GC_ has deleterious effect on GC survival

**Fig. 4:**
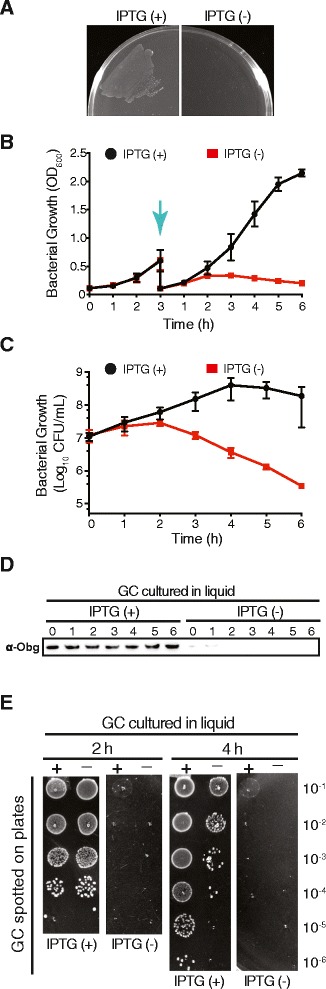
Obg_GC_ is essential for GC viability. **a** The FA1090 conditional *obg* knockout strain, P_*lac*_::*obg*_*GC*_, failed to grow when plated from freezer stocks onto GCB without (−) 100 μM IPTG, whereas abundant growth was observed on media supplemented with the inducer (+). **b**, **c** FA1090 cells carrying chromosomal P_*lac*_::*obg*_*GC*_ were collected from GCB agar plates supplemented with 100 uM IPTG, washed, divided, and grown in GCBL in the presence or absence of IPTG for 3 h. At this experimental time point (indicated by the blue arrow), the bacteria were harvested, washed again and growth was continued for 6 h in liquid media with (+) or without (−) IPTG, as indicated. Culture density was measured as Optical Density at OD_600_ (**b**). Cell viability was monitored every hour after the second inoculation by spotting serial dilutions onto GCB with IPTG (**c**). Experiments were performed in biological triplicates and means and SEM are presented. **d** Representative immunoblot showing Obg_GC_ levels over time in FA1090 P_*lac*_::*obg*_*GC*_ grown in the presence (+) and absence (−) of IPTG. The samples were collected every hour after back dilution (as indicated), matched by the same OD_600_ units, and whole cell lysates were probed with anti-Obg_GC_ antisera. **e** Cultures of FA1090 P_*lac*_::*obg*_*GC*_ grown in the liquid media in presence (+) and absence (−) of IPTG were serially diluted and spotted on GCB with (+) and without (−) the inducer. The 2- and 4-h time points from back dilution are shown

To examine whether Obg_GC_ plays a critical function in GC physiology, we used an allelic exchange approach and placed NGO1990 under the control of the isopropyl-β-D-thiogalactoside (IPTG)-inducible promoter, P_*lac*_, in its native chromosomal locus in GC FA1090. The resulting conditional knockout strain, FA1090 P_*lac*_::*obg*_GC_, failed to grow when inoculated directly from the freezer stocks onto the gonococcal base agar solid medium (GCB) lacking IPTG, whereas robust bacterial growth was observed in the presence of the inducer (Fig. 4a).

Subsequently, to examine the effect of Obg_GC_ depletion on GC viability over time, non-piliated and translucent colonies of FA1090 P_*lac*_::*obg*_*GC*_, harvested from GCB supplemented with 100 μM IPTG, were washed, suspended to the same OD_600_ of 0.1, divided, and cultured in gonococcal base liquid (GCBL) medium in the presence or absence of IPTG. After 2 h under repressive conditions, Obg_GC_ was still detectable by immunoblotting (Fig. 2a) and the bacterial proliferation rate was indistinguishable from the permissive condition (Fig. 4b). Similarly, depletion of *C. crescentus* Obg using the P_*xyl*_ promoter and repressive growth conditions (glucose instead of xylose) resulted in much lower but detectable levels of Obg even 12 h after a carbon shift [17].

**Fig. 5:**
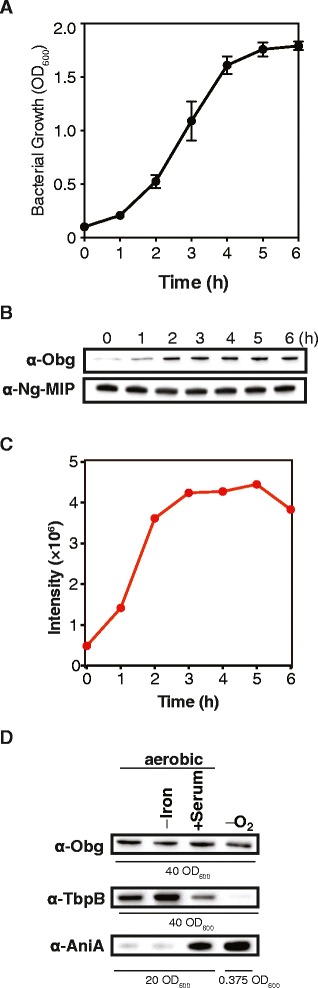
Expression of Obg_GC_. The growth of FA1090 (**a**) and Obg_GC_ amounts (**b**) were examined during regular aerobic conditions in GCBL by measurements of bacterial turbidity (OD_600_) and immunoblotting analyses of whole cell lysates every hour. The graph shows means with corresponding SEM from biological triplicate experiments. Samples were matched by equivalent OD_600_ units and representative immunoblots are shown. Immuoblotting with anti-Ng-MIP antisera was used as a loading control. **c** The immunoblot probed with anti-Obg_GC_ antisera was scanned and subjected to densitometric analysis. To quantify the intensity of the Obg_GC_ protein bands, the volume tool, local background subtraction, and linear regression methods were used. **d** The expression of Obg_GC_was assessed in whole cell lysates derived from GC cultured on GCB aerobically, in iron-limited conditions, in the presence of 7.5 % normal human sera, and anaerobically in the presence of nitrite as a terminal electron acceptor. Immunoblotting analyses with anti-TbpB and anti-AniA antisera were used as controls for iron-depleted [54] and anaerobic [55] growth conditions, respectively. Samples of whole-cell lysates were matched by the same OD_600_ units (40 or 20 as indicated) with the exception of detection of AniA during anaerobic growth conditions, where 0.375 OD_600_ units were used

Prolonged culturing of GC is not feasible because the bacteria undergo autolysis shortly after reaching stationary phase [48–52]. Therefore to ensure significant reduction in the amount of Obg_GC_, the conditional knockout strain FA1090 P_*lac*_::*obg*_*GC*_ was first treated as described above, cultures were collected 3 h after initial inoculation (indicated by an arrow in Fig. 4b), washed, and back diluted into fresh GCBL with or without the inducer. Culture density and bacterial viability, measured as Optical Density at 600 nm (OD_600,_ Fig. 4b) and Colony-Forming Units (CFUs, Fig. 4c), respectively, were monitored every hour. Growth kinetics of FA1090 P_*lac*_::*obg*^*GC*^ cultured in the presence of IPTG (Fig. 4a) closely followed the pattern observed in parental wild type strain (Fig. 5a). In contrast, under non-permissive conditions, the culture density and bacterial viability were decreased significantly (Fig. 4b and c, respectively), concomitant with the reduction in Obg_GC_ level (Fig. 4d). At 6 h of the experiment, the Obg_GC_-depleted culture contained on average 3.46 × 10^5^ live bacterial cells, whereas 1.87 × 10^8^ were present in permissive conditions (Fig. 4c). Further, FA1090 P_*lac*_::*obg*_*GC*_ grown in liquid media in the presence of IPTG was unable to survive upon plating on GCB lacking the inducer (Fig. 4e), confirming our prior observations (Fig. 4a).

Together, these studies demonstrate that Obg plays a pivotal function in GC physiology, as the depletion of Obg_GC_ caused loss of GC viability.

### Expression of Obg_GC_

The expression of Obg protein varies in different examined bacterial species. For instance, in *S. coelicolor* Obg is expressed in a growth-dependent manner with a sharp decline right after the beginning of aerial mycelium development and at the end of vegetative growth [28], whereas constant levels of Obg are maintained throughout the *C. crescentus* life cycle [30].

To examine the expression of Obg_GC_, wild type FA1090 was maintained under routine aerobic cultivation in GCBL. Bacterial proliferation was monitored by measurements of cell density at OD_600_ within 6 h of the experiment (Fig. 5a). Every hour GC samples were collected and the whole cell lysates were probed with anti-Obg_GC_ antisera. The same samples were also examined using antibodies against an unrelated protein, Ng-MIP (Fig. 5b) and by SDS-PAGE coupled with colloidal coomassie staining (Additional file 2: Figure S2B) as loading controls. Immunoblotting and densitometry analyses (Fig. 5b and c, respectively) showed that Obg_GC_ reached maximum expression in the early logarithmic phase of GC growth at OD_600_ ~ 0.5 (2 h from the start of the experiment), and remained constant until stationary phase.

We also asked whether the conditions that more closely resemble clinical infection, such as anoxia, iron deprivation and, in the event of disseminated infection, exposure to human serum [2, 53], influence expression of Obg_GC_. As expected, immunoblotting analysis showed increased levels of TbpB and AniA, which are well-recognized protein markers for iron-limited [54] and anaerobic [55] growth conditions, respectively (Fig. 5d). In contrast, the cellular concentrations of Obg_GC_ remained unaltered during growth of wild type FA1090 on GCB aerobically, in iron-limited conditions, in the presence of 7.5 % normal human sera, and anaerobically in the presence of nitrite as a terminal electron acceptor.

### Subcellular localization of Obg_GC_

The subcellular fractionation experiments showed that Obg was localized in the cytosol [35] and partially associated with the crude cell envelopes in *E. coli* [56], whereas in *S. coelicolor*, immunoelectron microscopy indicated that Obg was associated with the cytoplasmic membrane [28]. In addition, a growing number of reports show that different members of the Obg family cofractionate primarily with the 50S ribosomal subunit [17, 22, 32, 57]; however, in *Mycobacterium tuberculosis*, Obg is present in the 30S, 50S, and 70S ribosomal fractions [37].

**Fig. 6:**
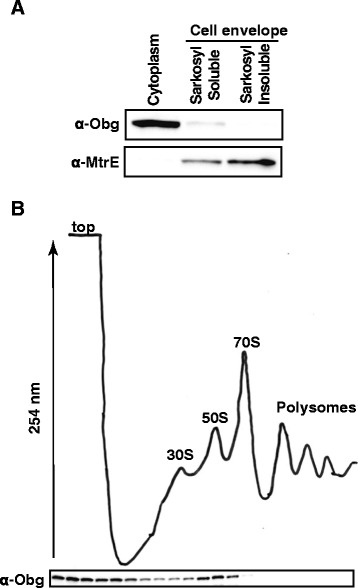
Obg is a primarily cytoplasmic protein that predominantly fractionates together with the 50S ribosomal particles. **a** Samples of isolated subproteome fractions (as indicated; 15 μg) obtained from wild type FA1090 cultured under regular aerobic laboratory growth conditions were separated in 4-20 % gradient gels and probed with either polyclonal rabbit anti-Obg_GC_ antisera or monoclonal mouse anti-MtrE antibodies. **b** FA1090 cell lysate was subjected to separation on a 10-47 % step sucrose gradient by ultracentrifugation at 174,000 × *g* for 4 h and monitored by UV absorbance at 254 nm. The positions of the 30S and 50S ribosomal subunits, the 70S monosomes, and polyribosomes are indicated. The level of Obg_GC_ in the collected fractions was detected by immunoblotting with polyclonal rabbit anti-Obg_GC_ antibodies. A representative polyribosome profile and immunoblot are shown

To examine the cellular localization of Obg_GC_, wild type FA1090 was cultured under standard laboratory conditions in GCBL and harvested at the mid-exponential phase of growth. The bacterial cells were lysed and the cell envelope proteins were separated from the cytosolic proteome by a sodium carbonate extraction procedure [58]. The crude cell envelopes were treated with sarkosyl to solubilize the inner membrane proteins, and the outer membrane was recovered by ultracentrifugation [59]. The same total amounts of purified subproteomes (15 μg) were separated by SDS-PAGE and probed with anti-Obg_GC_ antisera (Fig. 6a). The outer membrane protein, MtrE, which contains an extended periplasmic tunnel [60], was used as the cell envelope protein marker. As expected, MtrE was absent in the cytosol and enriched in the sarkosyl-insoluble fraction, whereas the vast majority of Obg_GC_ was present in the cytosolic protein fractions (Fig. 6a). A faint band of the same molecular weight was also detected in the cytoplasmic membrane. Many cytoplasmic proteins are repeatedly identified in different cell envelope proteomics studies and are often considered “contaminants” [61]. However, recent thorough sequential biochemical fractionations of *E. coli*, combined with mass spectrometry, demonstrated that many of these proteins, including Obg, form an actual peripheral inner membrane proteome linked via functional and/or structural oligomeric complexes [62].

Next, we addressed whether Obg_GC_ cofractionates with the ribosomes by ultracentrifugation of GC cell lysates through sucrose gradients and analysis of the polyribosomes profiles. Most of the cellular proteins accumulated at the top of the gradient followed by the peaks for small and large ribosomal subunits, the 70S monosomes, and the polyribosomes (Fig. 6b). Immunoblotting analysis with anti-Obg_GC_ antisera showed that under these conditions, the greatest amounts of Obg_GC_ were in 50S fractions and at the top of the gradient.

Based on these results, we conclude that Obg_GC_ is largely localized to the cytosol and primarily associates with the 50S ribosomal particle and not with the 70S monosomes or with translating ribosomes. Association of a part of the Obg_GC_ cellular pool with the cytoplasmic membrane may have functional implications, as Obg has been shown to be involved in key cellular processes such as ribosome maturation, DNA synthesis, cell division and morphology. Obg could be recruited to the membrane-bound complexes on demand, depending on the metabolic status of the bacterial cell.

### Obg_GC_ is expressed by contemporary clinical isolates of GC

Finally, the conservation of the predicted amino acid sequence of Obg_GC_ was assessed using the completed genome sequences of strains FA1090 (Gen Bank accession number AE004969) and NCCP11945 (Gen Bank accession number CP001050), as well as the draft genome sequences of 14 different GC strains (downloaded from the Broad Institute website http://www.broadinstitute.org/annotation/genome/neisseria_gonorrhoeae/MultiHome.html). These analyses demonstrated that Obg_GC_ is 100 % identical among 14 strains and has a single amino acid change in the GC isolates designated as DGI2 and PID18 (Additional file 3: Figure S3).

**Fig. 7:**
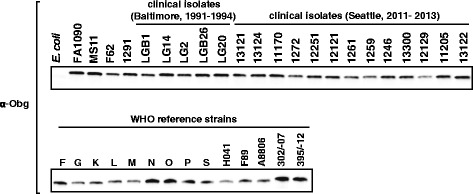
The polyclonal rabbit anti-Obg_GC_ antibodies cross-react with cell lysates of 36 diverse GC strains. Samples of whole-cell lysates derived from various GC isolates (as indicated) harvested from GCB were matched by equivalent OD_600_ units and resolved in 4-20 % Tris-glycine precast gels. The proteins were transferred onto nitrocellulose membrane and probed with polyclonal rabbit anti-Obg_GC_ antisera

Subsequently, to examine expression of Obg_GC_, a diversified panel of GC isolates was utilized. This panel included common laboratory strains MS11, F62, and 1291, as well as 32 strains isolated from different gonorrhea patients from distinct geographical areas and at different time points. The anti-Obg_GC_ antibodies detected a band of the same size in all examined GC isolates, albeit the level of expression varied between some strains (Fig. 7).

## Conclusions

Targeting essential proteins and critical cellular processes that are widely conserved remains an attractive avenue in antibacterial drug discovery programs. Compounds interfering with ribosome function and biogenesis and thus inhibiting different aspects of protein synthesis are among the most clinically useful antibiotics in spite of evolutionary conservation of bacterial and mitochondrial ribosomes [63].

Here, we show for the first time that Obg_GC_ is a GTPase essential for GC viability, mainly associated with the 50S large ribosomal subunit, abundant during different growth phases as well as under environmental conditions relevant to infection, and conserved in GC isolates. Together, these findings underscore the potential of Obg_GC_ as a target for the development of therapeutics against gonorrhea.

## Methods

### Bacterial strains, plasmids, and growth conditions

Strain of GC FA1090 [64] was primarily used in this study. Additionally, we employed: MS11 [65], 1291 [66], F62 [67], FA19 [68], isolates LGB1, LG14, LG20, and LG26 collected from two public health clinics in Baltimore between the years 1991–1994 [58], 13 strains derived from different patients seen at the Public Health–Seattle & King County STD clinic in 2011–2013 (Wierzbicki, et al., manuscript in preparation), as well as 14 WHO reference strains [69, 70]. Clinical isolates were kindly provided by Olusegun O. Soge, and King K. Holmes (Departments of Medicine and Global Health, University of Washington, Seattle, WA) and Magnus Unemo (Örebro University Hospital, Örebro, Sweden).

GC was maintained on GCB Medium Base agar plates with Kellogg’s supplements in 5 % CO_2_ atm at 37 °C or in GCBL Medium Base Broth containing Kellogg’s supplements and sodium bicarbonate (at a final concentration of 0.042 %) at 37 °C [48, 71, 72]. To achieve iron limited conditions, GCB without ferric nitrate in Kellogg’s Supplements and with deferoxamine mesylate salt (Desferal, Sigma) at 5 μM final concentration was utilized [73]. In addition, when stated in the text, GCB supplemented with 7.5 % normal human serum [74] was used to grow GC.

The *E. coli* strain NEB5α was used for genetic manipulations. Bacteria were streaked from −80 °C on Luria-Bertani (LB) agar supplemented with kanamycin (50 μg/mL) when needed. *E. coli* strains were cultured in LB medium at 37 °C. All media utilized in this study were purchased from Difco.

### Construction of recombinant wild type and mutated versions of Obg_GC_

All oligonucleotide primers designed and used in this study were synthesized by IDT DNA Technologies. Relevant restriction sites within primers are underlined. Recombinant N-His-Obg_GC_ and C-His-Obg_GC_ were generated by amplifying the *obg* gene from genomic DNA using two pairs of primers, respectively: obg-f 5’GAATTCCATATGAAATTCATCGACGAAGCAAAA3’ and obg-r 5’GAATTCAAGCTTTTACTCCGGCTTAAACACG3’; as well as obg-c-f 5’GACTCCATGGAATTCATCGACGAAGCAAAAATCG3’ and obg-c-r 5’GACTAAGCTTCTCCGGCTTAAACACGCC3’. The corresponding PCR products were digested with NdeI-HindIII or NcoI-HindIII (respectively) and cloned into similarly digested pET28a(+) to create plasmids pET-N-His-Obg_GC_ and pET-C-His-Obg_GC_. Mutagenesis of T192 and T193 residues into alanine were accomplished using the QuickChange II site directed mutagenesis kit (Agilent). Mutations were introduced using oligonucleotides: T192AT193A-f 5’GTTCGGATGCAGGGCGGCGAAGGGGTAGTTGG3’ and T192AT193A-r 5’CCAACTACCCCTTCGCCGCCCTGCATCCGAAC3’, and pET-N-His-Obg_GC_ as the DNA template. The presence of the desired mutations was confirmed by sequencing at the Center for Genome Research and Biocomputing at Oregon State University.

### Protein purification

The *E. coli* BL21(DE3) was used as a host for expression of all Obg_GC_ variants. Bacteria harboring N-His-Obg_GC_, C-His-Obg_GC_, or T192AT193A were cultured in LB at 37 °C until the cultures reached OD_600_ of ~ 0.5, and the expression of individual Obg_GC_ variants was induced by the addition of IPTG to a final concentration of 1 mM. Cells were pelleted 3 h after induction and resuspended in lysis buffer (20 mM Tris–HCl pH 8.0, 10 mM imidazole, 450 mM NaCl). The cell lysis was carried out by passing the suspension five times through a French pressure cell press at 12,000 psi. Bacterial debris was removed by centrifugation and the clarified crude cell extracts were loaded onto HisPur Cobalt Resin (Thermo) equilibrated with lysis buffer. Columns were subsequently washed with solution containing 20 mM Tris–HCl pH 8.0, 20 mM imidazole, and 450 mM NaCl. Proteins were eluted with the same buffer containing 250 mM imidazole. The eluate was dialyzed two times for an hour and overnight against 20 mM Tris–HCl pH 8.0. The purified proteins were concentrated using Microsep Advance Centrifugal Devices with 10 K molecular cutoff (PALL). Protein concentrations were determined using the Bradford method with a Protein Assay Kit (BioRad). Glycerol was added to purified proteins to a final concentration of 10 % and proteins were stored at −80 ° C until further use.

### Polyclonal rabbit anti-Obg_GC_ antisera

The polyclonal anti-Obg_GC_ antibodies were prepared by Pacific Immunology (Ramona, CA, USA) using the purified N-His-Obg_GC_, two New Zealand White rabbits, and a 13-week antibody production protocol approved by IACUC Animal Protocol #1, in a certified animal facility (USDA 93-R-283) and the NIH Animal Welfare Assurance Program (#A4182-01).

### Biochemical assays

The binding and hydrolysis of guanidine nucleotides were executed as described [26]. All fluorescence measurements were performed at 37 °C using a Synergy HT plate reader (BioTek). To determine the concentration of Mg^2+^ required for binding of mant-GTP or mant-GDP (Life Technologies), N-His-Obg_GC_ (2 μM) was incubated with either mant nucleotide (0.3 μM) and increasing concentrations of Mg^2+^ (from 0 to 10 mM) in binding buffer B containing 50 mM Tris–HCl pH 8.0, 50 mM KCl, 2 mM dithiothreitol, 10 μM ATP, and 10 % (wt/vol) glycerol. The experiments were repeated on three separate occasions and the data is presented as the percentage of maximal measured relative fluorescence units.

The ability to form GTP-and GDP-Obg complexes was studied using 0.3 μM mant-GTP or mant-GDP analogs in binding buffer B with or without 5 mM Mg^2+^, respectively, and 2 μM purified Obg_GC_ protein (N-His-Obg_GC_, T192AT193A, or C-His-Obg_GC_). These studies were carried out with at least eight biological replicates, and means with corresponding standard error of the mean (SEM) are reported.

To assess the GTP hydrolysis rate of Obg_GC_, N-His-Obg_GC_ or C-His-Obg_GC_ (16 μM) was prebound to 0.3 μM mant-GTP in binding buffer B supplemented with 5 mM MgCl_2._ The decrease in fluorescence associated with conversion of mant-GTP-Obg complexes into mant-GDP-Obg was recorded in 1 min intervals for 3 h. Data were fitted to a single exponential decay equation using GraphPad Prism 6.0f (Graph Pad Software). The single turnover rate constant and the half-life of hydrolysis were obtained from at least four independent experiments.

### Construction of conditional *obg*_*GC*_ mutant strain

The conditional *obg*_*GC*_ mutant strain, FA1090 P_*lac*_::*obg*_*GC*_, was constructed using a similar approach as we previously described [58]. Briefly, a gene encoding the Obg homolog in GC, Obg_GC_ (NGO1990), including an upstream region containing its indigenous ribosome-binding site (16 bp) was amplified with primers 5’CAAACAAGAGCATTTAATG3' and 5'AAGTTGGGCCGGCCTTACTCCGGCTTAAAC3'. To place the *obg*_*GC*_ under the control of the P_*lac*_ promoter, the resulting 1189 bp PCR product was digested with FseI and sub-cloned into ScaI-FseI digested pGGC4. This vector contains an IPTG-inducible promoter, which enables the controlled expression of a cloned gene [75]. The upstream region of *obg*_*GC*_ was amplified with primers 5'ACTAGTGAATTCGCCTTGCTGTCGCTTTG3' and 5'ATCGATGGTACCTTGGTTTTAAATAGGGTTTCAGGC3'. The obtained 531 bp product was digested with EcoRI and KpnI, and cloned into pUC18K [76], yielding the pUC18K-*obg*_*GC*_-up. Next, the DNA fragment containing *lacI* repressor gene, P_*lac*_ promoter and *obg*_*GC*_ gene carried on the pGCC4 was amplified with primers 5'ACTCAATAGGATCCTCACTGCCCGCTTTCCAG3' and 5'CATAAGCAGTCGACTTCAGACGGCGGAGACGGCGGTAATCAGG3'. The PCR product was purified, digested with BamHI-SalI and cloned into pUC18K [76], generating pUC18K-P_*lac*_::*obg*_*GC*_. This final construct encompassing nonpolar kanamycin resistance cassette *apha-3* [76] flanked by homologous regions for recombination and allelic exchange was used to introduce the mutation onto the FA1090 chromosome. The plasmid was linearized by digestion with NdeI and piliated bacteria were transformed with 0.1 μg of plasmid DNA in liquid media as described [77] with the exception that the growth media were supplemented with 100 μM IPTG. The resulting GC transformants were selected on GCB agar containing 40 μg/mL kanamycin and 100 μM IPTG. The FA1090 P_*lac*_::*obg*_*GC*_ clones were verified by PCR with primers 5’GCCTTGCTGTCGCTTTG3' and 5’ GGAGACGGCGGTAATCAGG3' and immunoblotting analysis with anti-Obg_GC_ antibodies.

### Obg_GC_ depletion studies

To assess the consequence of Obg_GC_ depletion on GC viability, the GC FA1090 P_*lac*_::*obg*_*GC*_ strain was plated from freezer stocks onto GCB supplemented with 100 μM IPTG. The following day, single nonpiliated colonies were passaged onto fresh GCB plates containing 100 μM IPTG and incubated approximately 18 h in 5 % CO_2_ atm at 37 °C. The bacteria were swabbed from plates, suspended in GCBL to OD_600_ of 0.1, and washed two times with GCBL that was pre-warmed to 37 °C to ensure removal of IPTG. Equal amounts of bacterial suspensions were divided into two flasks and cultured aerobically with and without 100 μM IPTG for 3 h at 37 °C. Subsequently, the cultures were diluted to OD_600_ of 0.1 into fresh GCBL with and without 100 μM IPTG. For measurements of OD_600_, bacterial viability (CFUs), and immunoblotting analysis, samples were withdrawn every hour and processed as described [58]. Experiments were performed in biological triplicates and mean values with corresponding SEM are presented.

### Subcellular fractionation

Non-piliated and translucent GC colonies of wild type FA1090 were swabbed from solid media, suspended in 500 mL of GCBL to OD_600_ of 0.1 and cultured at 37 °C with aeration (220 rpm) to OD_600_ of 0.6 - 0.8. Cells were harvested by centrifugation (20 min, 6000 × *g*) and the crude cell envelope fraction was separated from the cytosolic proteins using a sodium carbonate extraction procedure and subsequent ultracentrifugation steps [58]. The inner membrane proteins were solubilized using 2 % Sarkosyl in 20 mM Tris–HCl pH 7.5 according to the method described by Leuzzi et al. [59]. The Sarkosyl-insoluble outer membrane fractions were recovered by ultracentrifugation for 1 h at 100,000 × *g* and 4 °C. The pellet was suspended in PBS containing 1 % SDS. The total protein amount in each isolated subproteome fraction was assessed using a Protein Assay Kit (Bio Rad).

### Preparation of GC cell lysates for ribosome profiles

Wild type FA1090 bacteria were incubated in GCBL medium until the mid logarithmic phase of growth (OD_600_ ~ 0.5 to 0.7). Chloramphenicol was added to a final concentration of 100 μg/mL one minute before harvesting. Cells were harvested at 4,000 × g for 10 min at 4 °C and immediately frozen at −80 °C. After thawing on ice, bacteria were resuspended in 1 mL of lysis buffer comprised of 10 mM Tris–HCl pH 7.5, 10 mM MgCl_2,_ 30 mM NH_4_Cl, 100 μg/mL chloramphenicol [78]. Subsequently, an equal volume of glass beads (100 μm; Electron Microscopy Sciences) was added to the solution and bacterial cells were lysed by vortexing every minute for 10 min with a 1 min cooling interval on ice. Lysates were clarified by centrifugation for 15 min at 21,000 × *g* and 4 °C.

### Polyribosome fractionation

The isolated GC cell lysates in amounts corresponding to 15 OD_260_ units were overlaid on top of a 10 to 47 % step sucrose gradient as described previously [79]. Ribosomal subunits were separated by centrifugation in a Beckman SW41 rotor at 174,000 × *g* for 4 h and 4 ° C. Separated ribosomal subunits were fractionated using an Econo Pump and Econo UV Monitor (Bio-Rad). UV traces were recordered using a Model 1325 Econo Recorder (Bio-Rad) and 500 μL fractions were collected. Protein samples were precipitated with 15 % trichloroacetic acid. The resulting precipitates were solubilized in SDS loading buffer, and after separation in 10-20 % Criterion Tris-Tricine TGX (BioRad) acrylamide gel, all collected fractions were subjected to immunoblotting with anti-Obg_GC_ antisera as described below.

### SDS-PAGE and immunoblotting

Whole cell lysates were obtained from GC grown in GCBL with aeration and on GCB plates maintained under growth conditions as stated in the text. When bacterial colonies reached approximately the same size, all strains were harvested, suspended in pre-warmed GCBL, and the cell density was examined by OD_600_ measurement. Fractions containing either cytoplasmic, inner- or outer- membrane proteins (15 μg of proteins loaded per lane), ribosomal particles, or whole cell lysates matched by equivalent OD_600_ units, were prepared in SDS sample buffer in the presence of 50 mM dithiotreitol and separated in either 10-20 % Criterion Tris-Tricine TGX (BioRad) or 4-20 % Mini-PROTEAN TGX precast gels (Bio-Rad). The proteins were transferred onto 0.2 μm nitrocellulose membrane (Bio-Rad) using a Trans-blot Turbo (Bio-Rad). A solution of 5 % milk in phosphate buffered saline pH 7.0 (PBS, Li-Core) supplemented with 0.1 % Tween 20 (PBST) was used for blocking. Following 1 h of incubation, polyclonal rabbit antisera against Obg_GC_ (1:5,000), polyclonal anti-AniA antibodies (1:10,000; [58]),monoclonal mouse anti-MtrE antisera (1:10,000; a gift of Ann Jerse, Uniformed Services University, Bethesda), monoclonal mouse anti-Ng-MIP antibodies (1:10,000; a gift of Mariagrazia Pizza, Novartis Vaccines, Italy), or polyclonal rabbit anti-TbpB antisera (1:1,000; a gift of Cynthia Cornelissen, Virginia Commonwealth University, Richmond) diluted in PBST as indicated in parenthesis were added to the membranes. The horseradish peroxidase conjugate of goat anti-rabbit IgG antisera (BioRad) or goat anti-mouse IgG antibody (ThermoFisher Scientific), correspondingly, were utilized as secondary antibodies at 1:10,000 dilution. The reactions were developed using Clarity Western ECL-Substrate (BioRad) and a Chemi-Doc^TM^ MP System (BioRad) was used for western blot imaging.

### Densitometry

The immunoblot probed with anti-Obg_GC_ antisera was scanned using the ChemiDoc^TM^ system (BioRad) and subjected to densitometric analysis using Image Lab^TM^ 5.0 software (BioRad). To quantify the intensity of the Obg_GC_ protein bands, the volume tool (rectangle), local background subtraction, and linear regression were used.

### Statistical analyses

Statistical analyses were conducted using GraphPad Prism 6.0f (Graph Pad Software) and an unpaired Student’s *t-*test was used to analyze the data.

### Availability of supporting data

The data supporting the results of this article are included within its Additional files 1, 2 and 3: Figures S1-S3.

## Additional files

Additional file 1: Figure S1. Sequence alignment of Obg from *N. gonorrhoeae* and *N. meningitidis* with characterized members of Obg subfamily from various bacterial species. Identical residues are indicated by a star (*); G motifs (from G1 to G5) are underlined. Accession numbers of compared Obg homologs are as follows: *M. tuberculosis* (Mt) (CCE37910), *S. coelicolor* (Sc) (BAA13498), *D. radiodurans* (Dr) (NP_293810), *T. thermophilus* (Tt) (YP_145047), *B. subtilis* (Bs) (AAA22505), *C. crescentus* (Cc) (NP_419134), *N. gonorrhoeae* (Ng) (YP_209010), *N. meningitidis* (Nm) (NP_275074)*, E. coli* (Ec) (NP_417650), *V. cholerae* (Vc) (NP_230091). Additional file 2: Figure S2. Loading controls for immunoblotting experiments. Samples of whole-cell lysates were prepared for SDS-PAGE as described in the text, separated in precast gradient gels and the protein profiles were visualized using colloidal coomassie. Loaded OD_600_ units in individual experiments matched the corresponding samples used in immunoblotting analyses and are indicated below each gel. (A) Loading controls for immunoblotting experiment presented in Figure 2 A. (B) Loading controls for immunoblotting analysis shown in Figure 2 B. (C) Loading controls for experiment shown in Figure 5 B. (D) Loading controls for experiment in Figure 5 D for whole-cell lysates probed with anti-Obg and anti-TbpB antisera. (D) Loading controls for immunoblotting experiment with anti-AniA antisera presented in Figure 5 D. Additional file 3: Figure S3. Comparison of predicted amino acid sequences of Obg_GC_ between different GC isolates.

## Abbreviations

A: Absorbance
GC: *Neisseria gonorrhoeae*
GCB: Gonococcal base agar
GCBL: Gonococcal base liquid medium
OD: Optical density
IPTG: Isopropyl thio-β-D-galactopyranoside
PBS: Phosphate buffered saline
PBST: Phosphate buffered saline supplemented with 0.1 % Tween
SDS-PAGE: Sodium dodecyl sulfate polyacrylamide gel electrophoresis
SEM: Standard error of the mean
