# Supplementary material for: The Neisseria gonorrhoeae Obg protein is an essential ribosome-associated GTPase and a potential drug target
Source: BMC Microbiol. 2015 Jun 30;15:129. doi: 10.1186/s12866-015-0453-1 (PMC4487204; doi:10.1186/s12866-015-0453-1)
Supplement: Additional file 1: Figure S1. — Sequence alignment of Obg from N. gonorrhoeae and N. meningitidis with characterized members of Obg subfamily from various bacterial species. Identical residues are indicated by a star (*); G motifs (from G1 to G5) are underlined. Accession numbers of compared Obg homologs are as follows: M. tuberculosis (Mt) (CCE37910), S. coelicolor (Sc) (BAA13498), D. radiodurans (Dr) (NP_293810), T. thermophilus (Tt) (YP_145047), B. subtilis (Bs) (AAA22505), C. crescentus (Cc) (NP_419134), N. gonorrhoeae (Ng) (YP_209010), N. meningitidis (Nm) (NP_275074), E. coli (Ec) (NP_417650), V. cholerae (Vc) (NP_230091). [file 12866_2015_453_MOESM1_ESM.pdf]

Figure S1

```
Obg_Mt     MPRFVDRVVIHTRAGSGGNGCASVHREKFKPLGGPDGGNGGRGGSIVFVVDPQVHTLLDFHFRPHLTAASGKHGMGNNRDGAAGADLEVKVPEGTVVLDE-NGRLLADLVGAGTRFEAAA
Obg_Sc     MTTFVDRVELHVAAGNGGHGCASVHREKFKPLGGPDGGNGGRGGDVILTVDQSVTTLLDYHHSPHRKATNGKPGEGGNRSGKDGQDLVLPVPDGTVVLDG-AGNVLADLVGHGTSYVAAQ
Obg_Dr     -MAFRDVLNIEVAAGNGGDGSMSFHRAKYMEKGGPDGGHGGRGGSIILRAIEGVESLERLVGRRKFKAENGRYGEGRLRQGADGQDTYIDVPVGTTAFDEDSGKVIADLVNVGQEKVIAK
Obg_Tt     --MFQDVLVITVAAGRGGDGAVSFRREKFVPKGGPDGGDGGRGGSVYLRARGSVDSLSRLS-KRTYKAEDGEHGRGSQQHGRGGEDLVIEVPRGTRVFDADTGELLADLTEEGQTVLVAR
Obg_Bs     --MFVDQVKVYVKGGDGGNGMVAFRREKYVPKGGPAGGDGGKGGDVVFEVDEGLRTLMDFRYKKHFKAIRGEHGMSKNQHGRNADDMVIKVPPGTVVTDDDTKQVIADLTEHGQRAVIAR
Obg_Cc     -MKFLDQCKIYIRSGNGGGGSVSFRREKYIEYGGPDGGDGGRGGDVWIEAVEGLNTLIDYRYQQHFKAGTGVHGMGRARHGAAGEDVVLKVPVGTEVLEEDKETLIADLDHAGMRLLLAK
Obg_Ng     -MKFIDEAKIEVAAGKGGNGATSFRREKFVPRGGPDGGDGGKGGSVWAEADENTNTLVEYRFVKRYQAKNGEKGHGSDRYGAGADDIVLKMPVGTLIRDLDTDEIVADLTYHGQRVCLAK
Obg_Nm     -MKFIDEAKIEVAAGKGGNGATSFRREKFVPRGGPDGGDGGKGGSVWAEADENTNTLVEYRFVKRYQAKNGEKGHGSDRYGAGADDIVLKMPVGTLIRDLDTGETVADLTYHGQRVCLAK
ObgE_Ec    -MKFVDEASILVVAGDGGNGCVSFRREKYIPKGGPDGGDGGDGGDVWMEADENLNTLIDYRFEKSFRAERGQNGASRDCTGKRGKDVTIKVPVGTRVIDQGTGETMGDMTKHGQRLLVAK
Obg_Vc     -MKFVDEAVIKVQAGDGGNGVVSFWREKFVTNGGPDGGDGGDGGDVYMVADENLNTLIDYRFQRFYEAERGKNGGGGNCTGKSGKDKELRVPVGTRAVDIHTNEIIGEVAEHGKKVMIAK
             * *   :   .* ** *  :. * *:   *** **.** **.:    .      :*          *  *  * .    *  . *  : :* **   :      :.::   *     *

Obg_Mt     GGRGGLGNAALASRVRKAPGFALLGEKGQSRDLTLELKTVADVGLVGFPSAGKSSLVSAISAAKPKIADYPFTTLVPNLGVVSA-------GEHAFTVADVPGLIPGASRGRGLGLDFLR
Obg_Sc     GGRGGLGNAALASARRKAPGFALLGEPGDLQDIHLELKTVADVALVGYPSAGKSSLISVLSAAKPKIADYPFTTLVPNLGVVTA-------GETVYTVADVPGLIPGASQGKGLGLEFLR
Obg_Dr     GGLGGRGNSTFTSSTRQAPRFAELGTPGQKRRVRLELRLIADVGLVGYPNAGKSSLLAALSRANPAIADYPFTTLSPILGVVQREDEQGVSLDERFTMADIPGIIEGASEGKGLGLEFLR
Obg_Tt     GGAGGRGNMHFVSPTRQAPRFAEAGEEGEKRRLRLELMLIADVGLVGYPNAGKSSLLAAMTRAHPKIAPYPFTTLSPNLGVVEV------SEEERFTLADIPGIIEGASEGKGLGLEFLR
Obg_Bs     GGRGGRGNSRFATPANPAPQLSENGEPGKERYIVLELKVLADVGLVGFPSVGKSTLLSVVSSAKPKIADYHFTTLVPNLGMVET------DDGRSFVMADLPGLIEGAHQGVGLGHQFLR
Obg_Cc     GGNGGWGNLHFKGPVNQAPKYANPGQEGEERWIWLRLKLIADVGLVGLPNAGKSTFLAAASAAKPKIADYPFTTLTPNLGVVDL------SSSERFVLADIPGLIEGASEGAGLGTRFLG
Obg_Ng     GGKGGLGNIHFKSSVNRAPKQSTPGEEGETRSLQLELKVLADVGLLGMPNAGKSTLITAVSAARPKIANYPFTTLHPNLGVVRI------DENHSFVMADIPGLIEGAAEGAGLGHRFLK
Obg_Nm     GGKGGLGNIHFKSSVNRAPKQSTPGEEGEARSLQLELKVLADVGLLGMPNAGKSTLITAVSAARPKIANYPFTTLHPNLGVVRI------DENHSFVMADIPGLIEGAAEGAGLGHRFLK
ObgE_Ec    GGWHGLGNTRFKSSVNRTPRQKTNGTPGDKRELLLELMLLADVGMLGMPNAGKSTFIRAVSAAKPKVADYPFTTLVPSLGVVRM------DNEKSFVVADIPGLIEGAAEGAGLGIRFLK
Obg_Vc     GGWHGLGNARFKSSVNRSPRQKTLGTKGELRDIRLELLLLADVGMLGMPNAGKSTFIRAVSAAKPKVADYPFTTLVPSLGVVSV------LPEKSFVVADIPGLIEGAAEGAGLGIRFLK
           **  * **  :    . :*     *  *. : : *.*  :***.::* *..***::: . : *.* :* * **** * **:*                  :.:**:**:* ** .* ***  **
                                                   G1                     G2                                G3

Obg_Mt     HIERCAVLVHVVDCATAEPGRDPISDIDALETELACYTPTLQGDAALGDLAARPRAVVLNKIDVPEARELAEFVRDDIAQRGW----------------PVFCVSTATRENLQPLIFGLS
Obg_Sc     HVERCSVLVHVLDTATLESERDPLSDLDVIETELREY----------GGLDNRPRIVVLNKIDVPDGKDLAEMVRPDLEARGY----------------RVFEVSAVAHMGLRELSFALA
Obg_Dr     HISRTRLLVYVLDVT-----RNPVEELQQLQAELRAYDPS---------LLDNVALVALNKVELVEPDLAQMVEDELA----EQG-------------LPVLQVSAKEGTGLNTLRETLF
Obg_Tt     HIARTRVLLYVLDAA-----DEPLKTLETLRKEVGAYDPA---------LLRRPSLVALNKVDLLEEEAVKALADALA----REG-------------LAVLPVSALTGAGLPALKEALH
Obg_Bs     HIERTRVIVHVIDMSGLEGR-DPYDDYLTINQELSEYNLR---------LTERPQIIVANKMDMPEAA---ENLEAFKEKLTDDY---------------PVFPISAVTREGLRELLFEVA
Obg_Cc     HVERSATLIHLIDATQDDV----AGAYETIRGELEAYGDE---------LADKAEILALNKIDALDEETLAEKVAELEAVSGI----------------KPRLVSGVSGQGVTELLRAAY
Obg_Ng     HLSRTGLLLHVVDLAPFDETVNPAEEALAIINELRKYDEE---------LYGKPRWLVLNKLDMLDEEEARARTAAFLEAVGWDYPEPDDRFQFDMETPRLFQISALTHQGTQELVHQIN
Obg_Nm     HLSRTGLLLHVVDLAPFDETVNPAEEALAIINELRKYDEE---------LYGKPRWLVLNKLDMLDEEEAQTRTAAFLEAVGWDYPKPDDRFQFDMETPRLFQISALTHQGTQELVHQIN
ObgE_Ec    HLERCRVLLHLIDIDPIDGT-DPVENARIIISELEKYSQD---------LATKPRWLVFNKIDLLDKVEAEEKAKAIAEALGWED--------------KYYLISAASGLGVKDLCWDVM
Obg_Vc     HLERCRVLLHMIDIMPADQS-DPAHNALTIIDELEQYSEK---------LAKKPRWLVFNKVDLMSEEEADEIIQNIIDALAWEG--------------DYFKISAANRQGTKELCMKLA
           *: *   :::::*                 :  *:  *            *  .   :. **::  .                                   :*          *
                                                                G4                                              G5

Obg_Mt     QMISDYNAARPVAVPRRPV------------IRPIPVDDSGFTVEPDGHGGFVVSGARPE-RWIDQTNFDNDEAVGYLADRLARLGVEEELLRLGARSGCAVTIGE----MTFDWEPQTP
Obg_Sc     ELVATARAARPKEEATRIV------------IRPKAVDDAGFTVTREEDGLFRVRGEKPE-RWVRQTDFNNDEAVGYLSDRLNRLGVEDKLMKAGARNGDGVAIGPEDNAVVFDWEPSVT
Obg_Dr     QLLPEFELW-AQSNALEVEPDT----VVDEALQIVFREDAPAKGEGAPERVWEVHGGGFEERIV-RFSRYLEDAAEYLGNLFKRQGLYNALRRAGAREGDTVEIGT----FRFEYFDDEE
Obg_Tt     ALVRSTPPP-E-------MPKP----VPRKEVQA------GVEVVPVAEGVYEVRAPEVERYLA-RIKGDLMEAAGYLQEVFRRQGVEAALRAKGVRAGDLVRIGG----LEFEYIPEV-
Obg_Bs     NQLENT-PEFPLYDEEELTQNRVMYTME--------NEEVPFNITRDPDGVFVLSGDS-LERLFKMTDFSRDESVKRFARQMRGMGVDEALRERGAKDGDIIRLLE----FEFEFID---
Obg_Cc     KQVRIRRGDLEEEIDDD--E------------------DHVDETPGG----WTP-------------------------------------------------------------------
Obg_Ng     QYLAEK-KRIEAEKAEA--E-KAAANV-----------EIIEQQPKTDTGVFKPE-----------------------------------------------------------------
Obg_Nm     QYLTEK-KRIEAEKAEA--E-KAAANV-----------EIIEQQPKTDTGVFKPE-----------------------------------------------------------------
ObgE_Ec    TFIIEN-PVVQAEEAKQ--PEKVEFMWDDYHRQQLE--EIAEEDDEDWDDDWDEDDEEGVEFIYKR-----------------------------------------------------------
Obg_Vc     EFMDTL-PR-EAEEKTE--AEKVDFTWDYNHKDGLAGREVITEDDDDWDD-WDDEEDDGH-VIYVR-----D-----------------------------------------------------
             :                                                   :

Obg_Mt     AGEPVAMSGRGTDPRLDSNKRVGAAER--KAARSRRREHGDG----
Obg_Sc     AGAEML-GRRGEDHRFEAPRPAAQRRRDRDAERDEAQQEFDGFEPF
Obg_Dr     ----------------------------------------------
Obg_Tt     ----------------------------------------------
Obg_Bs     ----------------------------------------------
Obg_Cc     ----------------------------------------------
Obg_Ng     ----------------------------------------------
Obg_Nm     ----------------------------------------------
ObgE_Ec    ----------------------------------------------
Obg_Vc     ----------------------------------------------
```
