# Supplementary figures and images for: The Neisseria gonorrhoeae Obg protein is an essential ribosome-associated GTPase and a potential drug target
Source: BMC Microbiol. 2015 Jun 30;15:129. doi: 10.1186/s12866-015-0453-1 (PMC4487204; doi:10.1186/s12866-015-0453-1)

Figure S2

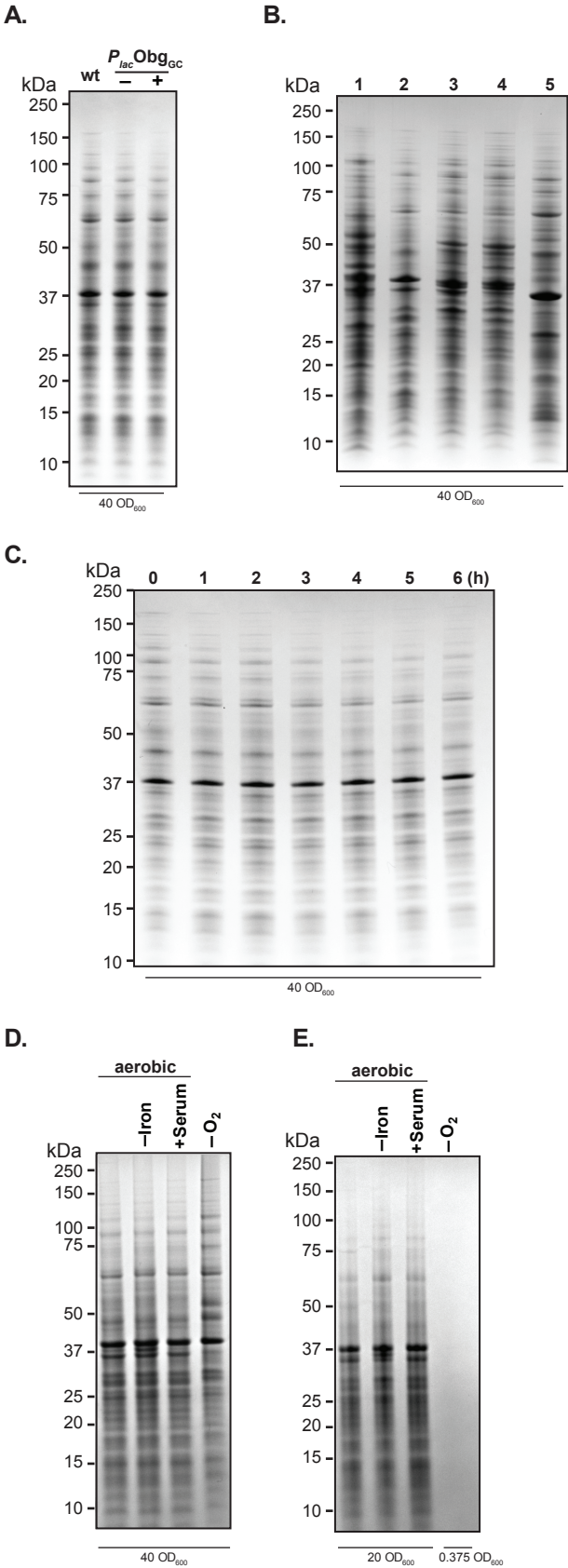

Supplement: Additional file 2: Figure S2. — Loading controls for immunoblotting experiments. Samples of whole-cell lysates were prepared for SDS-PAGE as described in the text, separated in precast gradient gels and the protein profiles were visualized using colloidal coomassie. Loaded OD600 units in individual experiments matched the corresponding samples used in immunoblotting analyses and are indicated below each gel. (A) Loading controls for immunoblotting experiment presented in Figure 2 A. (B) Loading controls for immunoblotting analysis shown in Figure 2 B. (C) Loading controls for experiment shown in Figure 5 B. (D) Loading controls for experiment in Figure 5 D for whole-cell lysates probed with anti-Obg and anti-TbpB antisera. (D) Loading controls for immunoblotting experiment with anti-AniA antisera presented in Figure 5 D. [file 12866_2015_453_MOESM2_ESM.pdf]
