# Supplementary material for: The Neisseria gonorrhoeae Obg protein is an essential ribosome-associated GTPase and a potential drug target
Source: BMC Microbiol. 2015 Jun 30;15:129. doi: 10.1186/s12866-015-0453-1 (PMC4487204; doi:10.1186/s12866-015-0453-1)
Supplement: Additional file 3: Figure S3. — Comparison of predicted amino acid sequences of ObgGC between different GC isolates. [file 12866_2015_453_MOESM3_ESM.pdf]

Supplemental File 3

```
FA1090          MKFIDEAKIEVAAGKGGNGATSFRREKFVPRGGPDGGDGGKGGSVWAEADENTNTLVEYR
NCCP11945       MKFIDEAKIEVAAGKGGNGATSFRREKFVPRGGPDGGDGGKGGSVWAEADENTNTLVEYR
FA19            MKFIDEAKIEVAAGKGGNGATSFRREKFVPRGGPDGGDGGKGGSVWAEADENTNTLVEYR
MS11            MKFIDEAKIEVAAGKGGNGATSFRREKFVPRGGPDGGDGGKGGSVWAEADENTNTLVEYR
F62             MKFIDEAKIEVAAGKGGNGATSFRREKFVPRGGPDGGDGGKGGSVWAEADENTNTLVEYR
1291            MKFIDEAKIEVAAGKGGNGATSFRREKFVPRGGPDGGDGGKGGSVWAEADENTNTLVEYR
PID332          MKFIDEAKIEVAAGKGGNGATSFRREKFVPRGGPDGGDGGKGGSVWAEADENTNTLVEYR
SK-92-679       MKFIDEAKIEVAAGKGGNGATSFRREKFVPRGGPDGGDGGKGGSVWAEADENTNTLVEYR
PID1            MKFIDEAKIEVAAGKGGNGATSFRREKFVPRGGPDGGDGGKGGSVWAEADENTNTLVEYR
SK-93-1035      MKFIDEAKIEVAAGKGGNGATSFRREKFVPRGGPDGGDGGKGGSVWAEADENTNTLVEYR
35/02           MKFIDEAKIEVAAGKGGNGATSFRREKFVPRGGPDGGDGGKGGSVWAEADENTNTLVEYR
DGI18           MKFIDEAKIEVAAGKGGNGATSFRREKFVPRGGPDGGDGGKGGSVWAEADENTNTLVEYR
FA6140          MKFIDEAKIEVAAGKGGNGATSFRREKFVPRGGPDGGDGGKGGSVWAEADENTNTLVEYR
PID24-1         MKFIDEAKIEVAAGKGGNGATSFRREKFVPRGGPDGGDGGKGGSVWAEADENTNTLVEYR
DGI2            MKFIDEAKIEVAAGKGGNGATSFRREKFVPRGGPDGGDGGKGGSVWAEADENTNTLVEYR
PID18           MKFIDEAKIEVAAGKGGNGATSFRREKFVPRGGPDGGDGGKGGSVWAEADENTNTLVEYR
                ************************************************************

FA1090          FVKRYQAKNGEKGHGSDRYGAGADDIVLKMPVGTLIRDLDTDEIVADLTYHGQRVCLAKG
NCCP11945       FVKRYQAKNGEKGHGSDRYGAGADDIVLKMPVGTLIRDLDTDEIVADLTYHGQRVCLAKG
FA19            FVKRYQAKNGEKGHGSDRYGAGADDIVLKMPVGTLIRDLDTDEIVADLTYHGQRVCLAKG
MS11            FVKRYQAKNGEKGHGSDRYGAGADDIVLKMPVGTLIRDLDTDEIVADLTYHGQRVCLAKG
F62             FVKRYQAKNGEKGHGSDRYGAGADDIVLKMPVGTLIRDLDTDEIVADLTYHGQRVCLAKG
1291            FVKRYQAKNGEKGHGSDRYGAGADDIVLKMPVGTLIRDLDTDEIVADLTYHGQRVCLAKG
PID332          FVKRYQAKNGEKGHGSDRYGAGADDIVLKMPVGTLIRDLDTDEIVADLTYHGQRVCLAKG
SK-92-679       FVKRYQAKNGEKGHGSDRYGAGADDIVLKMPVGTLIRDLDTDEIVADLTYHGQRVCLAKG
PID1            FVKRYQAKNGEKGHGSDRYGAGADDIVLKMPVGTLIRDLDTDEIVADLTYHGQRVCLAKG
SK-93-1035      FVKRYQAKNGEKGHGSDRYGAGADDIVLKMPVGTLIRDLDTDEIVADLTYHGQRVCLAKG
35/02           FVKRYQAKNGEKGHGSDRYGAGADDIVLKMPVGTLIRDLDTDEIVADLTYHGQRVCLAKG
DGI18           FVKRYQAKNGEKGHGSDRYGAGADDIVLKMPVGTLIRDLDTDEIVADLTYHGQRVCLAKG
FA6140          FVKRYQAKNGEKGHGSDRYGAGADDIVLKMPVGTLIRDLDTDEIVADLTYHGQRVCLAKG
PID24-1         FVKRYQAKNGEKGHGSDRYGAGADDIVLKMPVGTLIRDLDTDEIVADLTYHGQRVCLAKG
DGI2            FVKRYQAKNGEKGHGSDRYGAGADDIVLKMPVGTLIRDLDTDEIVADLTYHGQRVCLAKG
PID18           FVKRYQAKNGEKGHGSDRYGAGADDIVLKMPVGTLIRDLDTDEIVADLTYHGQRVCLAKG
                ************************************************************

FA1090          GKGGLGNIHFKSSVNRAPKQSTPGEEGETRSLQLELKVLADVGLLGMPNAGKSTLITAVS
NCCP11945       GKGGLGNIHFKSSVNRAPKQSTPGEEGETRSLQLELKVLADVGLLGMPNAGKSTLITAVS
FA19            GKGGLGNIHFKSSVNRAPKQSTPGEEGETRSLQLELKVLADVGLLGMPNAGKSTLITAVS
MS11            GKGGLGNIHFKSSVNRAPKQSTPGEEGETRSLQLELKVLADVGLLGMPNAGKSTLITAVS
F62             GKGGLGNIHFKSSVNRAPKQSTPGEEGETRSLQLELKVLADVGLLGMPNAGKSTLITAVS
1291            GKGGLGNIHFKSSVNRAPKQSTPGEEGETRSLQLELKVLADVGLLGMPNAGKSTLITAVS
PID332          GKGGLGNIHFKSSVNRAPKQSTPGEEGETRSLQLELKVLADVGLLGMPNAGKSTLITAVS
SK-92-679       GKGGLGNIHFKSSVNRAPKQSTPGEEGETRSLQLELKVLADVGLLGMPNAGKSTLITAVS
PID1            GKGGLGNIHFKSSVNRAPKQSTPGEEGETRSLQLELKVLADVGLLGMPNAGKSTLITAVS
SK-93-1035      GKGGLGNIHFKSSVNRAPKQSTPGEEGETRSLQLELKVLADVGLLGMPNAGKSTLITAVS
35/02           GKGGLGNIHFKSSVNRAPKQSTPGEEGETRSLQLELKVLADVGLLGMPNAGKSTLITAVS
DGI18           GKGGLGNIHFKSSVNRAPKQSTPGEEGETRSLQLELKVLADVGLLGMPNAGKSTLITAVS
FA6140          GKGGLGNIHFKSSVNRAPKQSTPGEEGETRSLQLELKVLADVGLLGMPNAGKSTLITAVS
PID24-1         GKGGLGNIHFKSSVNRAPKQSTPGEEGETRSLQLELKVLADVGLLGMPNAGKSTLITAVS
DGI2            GKGGLGNIHFKSSVNRAPKQSTPGEEGETRSLQLELKVLADVGLLGMPNAGKSTLITAVS
PID18           GKGGLGNIHFKSSVNRAPKQSTPGEEGETRSLQLELKVLADVGLLGMPNAGKSTLITAVS
                ************************************************************

FA1090          AARPKIANYPFTTLHPNLGVVRIDENHSFVMADIPGLIEGAAEGAGLGHRFLKHLSRTGL
NCCP11945       AARPKIANYPFTTLHPNLGVVRIDENHSFVMADIPGLIEGAAEGAGLGHRFLKHLSRTGL
FA19            AARPKIANYPFTTLHPNLGVVRIDENHSFVMADIPGLIEGAAEGAGLGHRFLKHLSRTGL
MS11            AARPKIANYPFTTLHPNLGVVRIDENHSFVMADIPGLIEGAAEGAGLGHRFLKHLSRTGL
F62             AARPKIANYPFTTLHPNLGVVRIDENHSFVMADIPGLIEGAAEGAGLGHRFLKHLSRTGL
1291            AARPKIANYPFTTLHPNLGVVRIDENHSFVMADIPGLIEGAAEGAGLGHRFLKHLSRTGL
PID332          AARPKIANYPFTTLHPNLGVVRIDENHSFVMADIPGLIEGAAEGAGLGHRFLKHLSRTGL
SK-92-679       AARPKIANYPFTTLHPNLGVVRIDENHSFVMADIPGLIEGAAEGAGLGHRFLKHLSRTGL
```

```
PID1            AARPKIANYPFTTLHPNLGVVRIDENHSFVMADIPGLIEGAAEGAGLGHRFLKHLSRTGL
SK-93-1035      AARPKIANYPFTTLHPNLGVVRIDENHSFVMADIPGLIEGAAEGAGLGHRFLKHLSRTGL
35/02           AARPKIANYPFTTLHPNLGVVRIDENHSFVMADIPGLIEGAAEGAGLGHRFLKHLSRTGL
DGI18           AARPKIANYPFTTLHPNLGVVRIDENHSFVMADIPGLIEGAAEGAGLGHRFLKHLSRTGL
FA6140          AARPKIANYPFTTLHPNLGVVRIDENHSFVMADIPGLIEGAAEGAGLGHRFLKHLSRTGL
PID24-1         AARPKIANYPFTTLHPNLGVVRIDENHSFVMADIPGLIEGAAEGAGLGHRFLKHLSRTGL
DGI2            AARPKIANYPFTTLHPNLGVVRIDENHSFVMADIPGLIEGAAEGAGLGHRFLKHLSRTGL
PID18           AARPKIANYPFTTLHPNLGVVRIDENHSFVMADIPGLIEGAAEGAGLGHRFLKHLSRTGL
                ************************************************************

FA1090          LLHVVDLAPFDETVNPAEEALAIINELRKYDEELYGKPRWLVLNKLDMLDEEEARARTAA
NCCP11945       LLHVVDLAPFDETVNPAEEALAIINELRKYDEELYGKPRWLVLNKLDMLDEEEARARTAA
FA19            LLHVVDLAPFDETVNPAEEALAIINELRKYDEELYGKPRWLVLNKLDMLDEEEARARTAA
MS11            LLHVVDLAPFDETVNPAEEALAIINELRKYDEELYGKPRWLVLNKLDMLDEEEARARTAA
F62             LLHVVDLAPFDETVNPAEEALAIINELRKYDEELYGKPRWLVLNKLDMLDEEEARARTAA
1291            LLHVVDLAPFDETVNPAEEALAIINELRKYDEELYGKPRWLVLNKLDMLDEEEARARTAA
PID332          LLHVVDLAPFDETVNPAEEALAIINELRKYDEELYGKPRWLVLNKLDMLDEEEARARTAA
SK-92-679       LLHVVDLAPFDETVNPAEEALAIINELRKYDEELYGKPRWLVLNKLDMLDEEEARARTAA
PID1            LLHVVDLAPFDETVNPAEEALAIINELRKYDEELYGKPRWLVLNKLDMLDEEEARARTAA
SK-93-1035      LLHVVDLAPFDETVNPAEEALAIINELRKYDEELYGKPRWLVLNKLDMLDEEEARARTAA
35/02           LLHVVDLAPFDETVNPAEEALAIINELRKYDEELYGKPRWLVLNKLDMLDEEEARARTAA
DGI18           LLHVVDLAPFDETVNPAEEALAIINELRKYDEELYGKPRWLVLNKLDMLDEEEARARTAA
FA6140          LLHVVDLAPFDETVNPAEEALAIINELRKYDEELYGKPRWLVLNKLDMLDEEEARARTAA
PID24-1         LLHVVDLAPFDETVNPAEEALAIINELRKYDEELYGKPRWLVLNKLDMLDEEEARARTAA
DGI2            LLHVVDLAPFDETVNPAEEALAIINELRKYDEELYGKPRWLVLNKLDMLDEEEARARTAA
PID18           LLHVVDLAPFDETVNPAEEALAIINELRKYDEELYGKPRWLVLNKLDMLDEEEARARTAA
                ************************************************************

FA1090          FLEAVGWDYPEPDDRFQFDMETPRLFQISALTHQGTQELVHQINQYLAEKKRIEAEKAEA
NCCP11945       FLEAVGWDYPEPDDRFQFDMETPRLFQISALTHQGTQELVHQINQYLAEKKRIEAEKAEA
FA19            FLEAVGWDYPEPDDRFQFDMETPRLFQISALTHQGTQELVHQINQYLAEKKRIEAEKAEA
MS11            FLEAVGWDYPEPDDRFQFDMETPRLFQISALTHQGTQELVHQINQYLAEKKRIEAEKAEA
F62             FLEAVGWDYPEPDDRFQFDMETPRLFQISALTHQGTQELVHQINQYLAEKKRIEAEKAEA
1291            FLEAVGWDYPEPDDRFQFDMETPRLFQISALTHQGTQELVHQINQYLAEKKRIEAEKAEA
PID332          FLEAVGWDYPEPDDRFQFDMETPRLFQISALTHQGTQELVHQINQYLAEKKRIEAEKAEA
SK-92-679       FLEAVGWDYPEPDDRFQFDMETPRLFQISALTHQGTQELVHQINQYLAEKKRIEAEKAEA
PID1            FLEAVGWDYPEPDDRFQFDMETPRLFQISALTHQGTQELVHQINQYLAEKKRIEAEKAEA
SK-93-1035      FLEAVGWDYPEPDDRFQFDMETPRLFQISALTHQGTQELVHQINQYLAEKKRIEAEKAEA
35/02           FLEAVGWDYPEPDDRFQFDMETPRLFQISALTHQGTQELVHQINQYLAEKKRIEAEKAEA
DGI18           FLEAVGWDYPEPDDRFQFDMETPRLFQISALTHQGTQELVHQINQYLAEKKRIEAEKAEA
FA6140          FLEAVGWDYPEPDDRFQFDMETPRLFQISALTHQGTQELVHQINQYLAEKKRIEAEKAEA
PID24-1         FLEAVGWDYPEPDDRFQFDMETPRLFQISALTHQGTQELVHQINQYLAEKKRIEAEKAEA
DGI2            FLEAVGWDYPEPDDRFQFDMETPRLFQISALTHQGTQELVHQINQYLAEKKRIEAKKAEA
PID18           FLEAVGWDYPEPDDRFQFDMETPRLFQISALTHQGTQELVHQINQYLAEKKRIEAKKAEA
                *******************************************************:****

FA1090          EKAAANVEIIEQQPKTDTGVFKPE
NCCP11945       EKAAANVEIIEQQPKTDTGVFKPE
FA19            EKAAANVEIIEQQPKTDTGVFKPE
MS11            EKAAANVEIIEQQPKTDTGVFKPE
F62             EKAAANVEIIEQQPKTDTGVFKPE
1291            EKAAANVEIIEQQPKTDTGVFKPE
PID332          EKAAANVEIIEQQPKTDTGVFKPE
SK-92-679       EKAAANVEIIEQQPKTDTGVFKPE
PID1            EKAAANVEIIEQQPKTDTGVFKPE
SK-93-1035      EKAAANVEIIEQQPKTDTGVFKPE
35/02           EKAAANVEIIEQQPKTDTGVFKPE
DGI18           EKAAANVEIIEQQPKTDTGVFKPE
FA6140          EKAAANVEIIEQQPKTDTGVFKPE
PID24-1         EKAAANVEIIEQQPKTDTGVFKPE
DGI2            EKAAANVEIIEQQPKTDTGVFKPE
PID18           EKAAANVEIIEQQPKTDTGVFKPE
                ************************
```
